# STA-MCA Bypass in Carotid Stenosis after Radiosurgery for Cavernous Sinus Meningioma

**DOI:** 10.3390/cancers13102420

**Published:** 2021-05-17

**Authors:** Marco Vincenzo Corniola, Marton König, Torstein Ragnar Meling

**Affiliations:** 1 Department of Neurosurgery, Geneva University Hospitals, 1205 Geneva, Switzerland; torstein.meling@hcuge.ch; 2 Faculty of Medicine, University of Geneva, 1205 Geneva, Switzerland; 3 Department of Neurology, Oslo University Hospital, N-0424 Oslo, Norway; m.s.s.konig@medisin.uio.no

**Keywords:** meningioma, neurosurgery, radiosurgery, cerebral ischemia, cerebral bypass, cavernous sinus, review

## Abstract

**Simple Summary:**

We present the case of a patient with a radiation-induced internal carotid artery stenosis after stereotactic radiosurgery for cavernous sinus meningioma. Our case presented with symptomatic vascular insufficiency two years after treatment. We carried out a review of the literature searching for vascular and non-vascular complications following the treatment of cavernous sinus meningiomas with radiosurgery or radiotherapy. As a result, two cases of stroke and one case of asymptomatic stenosis of the internal carotid artery were described, aside from non-vascular complications. We were able to conclude that radiosurgery and radiotherapy carry fewer complications than open surgery, with similar rates of tumor control. Altogether, our case and the associated review emphasize the importance of a multidisciplinary, extended follow-up of irradiated cavernous sinus meningiomas.

**Abstract:**

Background: Cavernous sinus meningiomas (CSM) are mostly non-surgical tumors. Stereotactic radiosurgery (SRS) or radiotherapy (SRT) allow tumor control and improvement of pre-existing cranial nerve (CN) deficits. We report the case of a patient with radiation-induced internal carotid artery (ICA) stenosis. We complete the picture with a review of the literature of vascular and non-vascular complications following the treatment of CSMs with SRS or SRT. Methods: After a case description, a systematic literature review is presented, according to the Preferred Reporting Items for Systematic Reviews and Meta-Analyses 2015 guidelines. Results: 115 abstracts were screened and 70 titles were retained for full-paper screening. A total of 58 articles did not meet the inclusion criteria. There were 12 articles included in our review, with a follow-up ranging from 33 to 120 months. Two cases of post-SRT ischemic stroke and one case of asymptomatic ICA stenosis were described. Non-vascular complications were reported in all articles. Conclusion: SRS and SRT carry fewer complications than open surgery, with similar rates of tumor control. Our case shows the importance of a follow-up of irradiated CSMs not only by a radio-oncologist, but also by a neurosurgeon, illustrating the importance of multidisciplinary management of CSMs.

## 1. Introduction

Cavernous sinus meningioma (CSM) is a rare subset of meningiomas and constitutes circa 1% of all intracranial meningiomas [1], representing the most frequent tumor in the parasellar region [2]. They are mostly World Health Organization (WHO) grade I lesions, with a meningiothelial histology [3]. Despite their fully benign nature, their evolution is unpredictable and irregular, and their clinical symptoms do not correlate with the initial tumor size and growth rate [4].

Purely intracavernous meningiomas are generally not considered for surgery, at least not as a stand-alone therapy, due to their proximity to cranial nerves (CNs), vascular structures, as well as the pituitary gland [4]. Aside from the challenging location, the consistency of CSMs may render their surgical resection very difficult because they can vary from a soft, easy-to-aspire consistency to a firm, trabeculous, almost-impossible-to-resect lesions. Alternatively, stereotactic radiosurgery (SRS) or radiotherapy (SRT) have been shown to be effective in the management of CSMs over the past two decades [5,6,7,8,9,10], either as first-line or adjuvant therapies.

SRS and SRT have been shown to allow excellent local tumor control and neurological improvement of pre-existing cranial nerve (CN) deficits [11]. However, long-term risks of such treatments, such as radiation-induced stenosis of the internal carotid artery (ICA) [12,13,14,15], are rare and poorly described. Other complications, such as CN function impairment, including visual loss and diplopia, should be considered when it comes to suggesting SRS or SRT to a patient with CSM.

Our aim was to report the case of a patient with radiation-induced ICA stenosis requiring a superior temporal artery–middle cerebral artery (STA-MCA) bypass as an example of a severe post-interventional complication of SRS. On the basis of this case, we complete the picture with a review of the literature of vascular and non-vascular complications following treatment of CSMs with SRS or SRT. Finally, the pros and cons of non-surgical treatment of CSMs are discussed, as is the multidisciplinary approach, management, and follow-up of these very challenging lesions.

## 2. Case Description

A 53-year-old female was referred to our department after having presented multiples episodes of dysphasia and short episodes (few minutes) of motor weakness in her right arm and leg, followed by right facial hypoesthesia when exercising. These were attributed to recurrent transient ischemic attacks (TIAs), and the symptoms suggested a cerebral hypoperfusion affecting the left frontal lobe. The patient’s medical history revealed that she had received SRS two years earlier for a pauci-symptomatic CSM (left-sided abducens palsy) that encased the left ICA without narrowing it.

**Figure 1:**
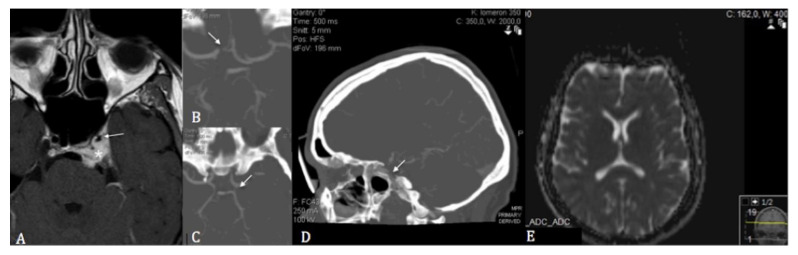
(**A**) T1 gadolinium-enhanced axial cerebral magnetic resonance imaging showing the encasement of the left internal carotid artery (asterisk) with subsequent stenosis of its cavernous segment (white arrow) following stereotactic radiosurgery. (**B**) Axial computed tomographic angiography showing the hypoplastic pre-and post-communicating segments of the right anterior cerebral artery. (**C**) Axial computed tomographic angiography showing the left hypoplastic posterior communicating artery. (**D**) Sagittal computed tomographic angiography showing the stenosis of the cavernous segment of the left internal carotid artery. (**E**) Diffusion-weighted sequences showing no restriction of diffusion on admission magnetic resonance imaging.

**Figure 2:**
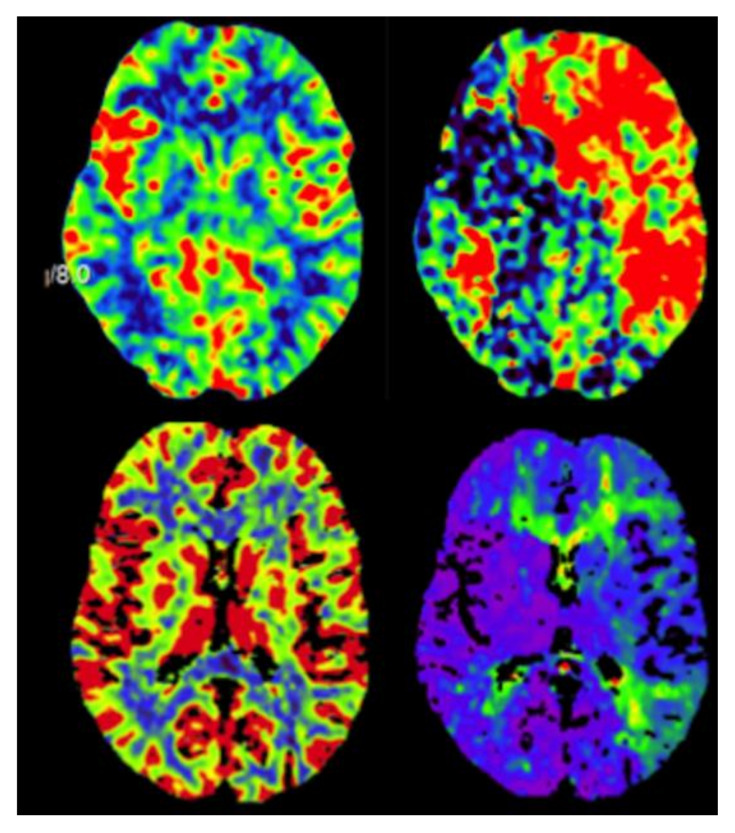
Perfusions imaging studies showing pre-operative cerebral blood volume and time-to-peak (top) and post-operative cerebral blood volume and time-to-peak (bottom).

Upon admission, her neurological examination was normal. The patient had no signs of infarcts on magnetic resonance imaging (MRI; Figure 1), while stenosis of the cavernous segment of the left ICA was observed (Figure 1), which was later confirmed on digital subtraction angiography (DSA). The A1 segment of the right anterior cerebral artery (ACA) and the left posterior communicating artery (PCOM) were hypoplastic (Figure 1). Perfusion MRI indicated a partially compensated flow through the left cerebral hemisphere related to the MCA and ACA territories, with a delayed mean transit time (MTT), marginally elevated cerebral blood volume (CBV), reduced cerebral blood flow (CBF), and delayed time-to-peak (Figure 2). Transcranial doppler ultrasound (TCD) showed reduced flow and pulsatility in the left MCA, reversed flow in the left A1 segment, and increased flow in the P1 segment of the left posterior cerebral artery (PCA). Intracranial pulsatility was detected in the external carotid artery, supporting the theory of existing secondary collaterals.

As the patient was pauci-symptomatic and not eager to have an intervention, she opted for conservative treatment, and a prophylactic treatment with acetylsalicylic acid (ASA) (75 mg/day) and statins (simvastatin 40 mg/day) was started. After three months, she continued to have mild TIAs with dysphasia on exercise, and repeat MRI perfusion studies did not show any development of secondary collaterals.

**Figure 3:**
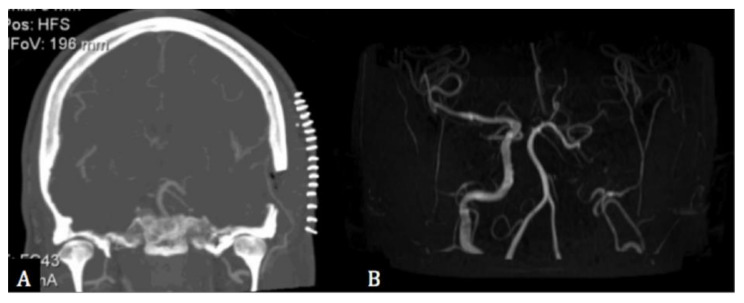
(**A**) Post-operative coronal computed tomographic angiography showing the bypass between the superior temporal and the middle cerebral arteries. (**B**) 3-years post-operative magnetic resonance angiography showing the occlusion of the left internal carotid artery and the left middle cerebral artery supplied by the bypass.

Eventually, a STA-MCA bypass was offered to the patient, connecting the left STA with the M4 segment of the ipsilateral MCA (Figure 3). A low-flow was chosen over high-flow bypass because the perfusion sequences were not severely impaired, and time-to-peak sequences showed a clear asymmetry between the right and left Sylvian territories. Post-operatively, the patient had an uneventful recovery and started prophylactic treatment with clopidogrel (75 mg/day) in addition to ASA. Post-operative TCDs showed a gradually increased flow through the bypass, and MR-angiography three years after treatment showed an occlusion of the left ICA and a left open MCA supplied through the bypass (Figure 3). The patient had no hemodynamic events during the follow-up period of 6 years.

## 3. Materials and Methods

### 3.1. Search Strategy and Study Selection

The systematic literature review was conducted according to the Preferred Reporting Items for Systematic Reviews and Meta-Analyses (PRISMA-P) 2015 guidelines [16]. Registration was not performed. On 20 March 2021, we performed a search of the literature in Embase, Cochrane Library, PubMed, Google Scholar, and Web of Science. The following Medical Subject Heading (MeSH) terms were used: (cavernous sinus meningioma AND radiotherapy AND post-operative carotid stenosis) OR (cavernous sinus meningioma AND radiotherapy AND complications), OR (cavernous sinus meningioma AND bypass), resulting in a list of 115 articles.

The following inclusion criteria were used: (1) peer-reviewed research articles, retrospective or prospective, on cavernous sinus meningiomas (exclusively) and post-radiosurgery/radiotherapy (either single-session or fractionated) carotid stenosis requiring/not requiring surgical bypass; (2) radiologically suspected CSM; (3) number of cases >5 patients; (4) studies written in English, French, German, or Italian language.

**Figure 4:**
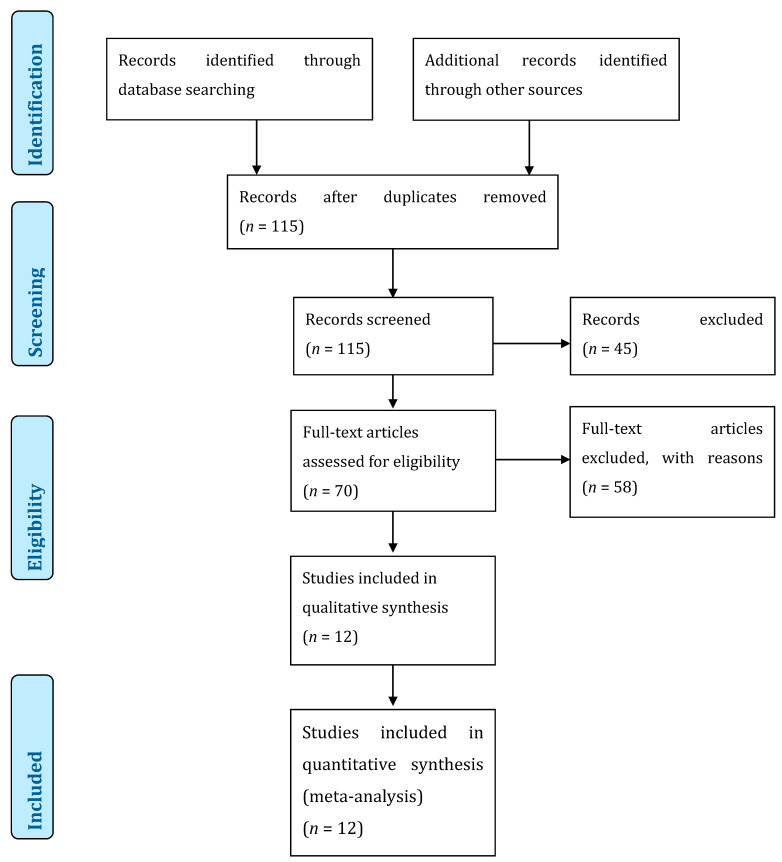
Preferred Reporting Items for Systematic Reviews and Meta-Analyses (PRISMA) flow diagram of the literature review.

Exclusion criteria were: other tumors than CSMs, vascular complications other than carotid stenosis, other treatment than RS or RT, publications other than original reports, redundant data of a single dataset. Editorials, letters, review articles, and case reports were excluded. The authors screened titles and abstracts of all the articles independently and all the relevant full-text copies were acquired (Figure 4).

### 3.2. Data Collection

The following data items were considered: (1) study ID; (2) study characteristics (author, year, country, prospective or retrospective study); (3) patient demographics; (4) sample size; (5) management of the stenosis (surgical versus non-surgical). If necessary, a consensus was reached by the authors through discussions with the senior author (TRM).

### 3.3. Risk of Bias and Quality of Study

The accepted articles were independently graded according to the Newcastle–Ottawa Quality Assessment Scale for quality assessment of non-randomized studies [17] (Table 1).

## 4. Results

### 4.1. Articles Included

In total, 115 abstracts were screened, and 70 titles were retained for full-paper screening. A total of 58 articles did not present enough data to meet the inclusion criteria. There were 12 articles included in our review (Figure 4) [4,7,8,9,10,18,19,20,21,22,23,24]. After noticing that some articles came from the same institution, we decided to renounce doing a meta-analysis because of the bias induced by duplicates between studies. According to the Newcastle-Ottawa Quality Assessment form for non-randomized studies, all studies were graded as fair (Table 2).

### 4.2. Follow-Up

A follow-up was reported in all studies, ranging from 33 to 120 months (Table 2).

### 4.3. Vascular Complications

With respect to reported complications, Pollock et al. [10] reported two cases of post-SRT ischemic stroke, while Correa et al. [22] reported one case of asymptomatic ICA stenosis (Table 1).

### 4.4. Non-Vascular Complications

All the articles reported non-vascular complications to SRS/SRT, such as trigeminal dysfunction, pituitary insufficiency, diplopia, radiation necrosis, brain edema, and radio-induced meningioma (Table 1).

## 5. Discussion

We present an illustrative case of a patient with a CSM treated by SRS who presented with a post-interventional symptomatic ICA stenosis requiring surgical management by an STA-MCA bypass. We complete the picture with a systematic review of the literature focused on vascular and non-vascular complications following SRS/SRT for CSM. To our knowledge, this is the first report of symptomatic post-irradiation stenosis undergoing surgical revascularization treatment.

### 5.1. Open Surgery for CSMs

During the infancy of skull base surgery, aggressive removal of CSMs was often attempted. However, due to the very complex location and the unpredictable consistency of the tumor, surgery has been shown to be mostly unsuccessful in achieving gross total resection and has been marked by high complication rates. Today, a more conservative policy favoring preservation of function and quality of life, rather than the radical resection at all costs, is often favored [25,26]. If surgery is indicated, CSMs can be approached surgically either trans-cranially or trans-nasally. Exophytic CSMs that need surgical debulking before SRS/SRT are best approached transcranially, whereas debulking of purely intracavernous CSMs are best performed transnasally.

A cornerstone publication came from Sindou et al. [27], who reported the long-term outcome (mean follow-up: 8.3 years) of *N* = 100 patients undergoing surgical resection of CSMs, either as a stand-alone therapy or with adjuvant SRS or SRT. The mortality rate was 5%; 2% of the patients had symptomatic ischemic sequelae, while onset or aggravation of a pre-existing visual loss, diplopia, or trigeminal dysfunction occurred in 19%, 29%, and 24% of patients, respectively [27]. Altogether, these results indicate that while satisfactory tumor control can be achieved with surgery, severe and functionally impairing complications are common and more frequent than with radiation therapy [27]. In the same vein, Shaffrey et al. [28] showed that CSMs encasing the ICA not only narrow the lumen but tend to infiltrate the vessel wall; in that perspective, surgery seems dangerous in terms of attempted radical resection of the lesion.

### 5.2. SRS and SRT as Effective—But Not Trivial—Treatments

Pollock et al. [10] reviewed the factors associated with local control and complications after single-fraction SRS. After 5 and 10 years, the local control rates were 99% and 93%, respectively. However, permanent complications were seen in up to 12% of the patients and included trigeminal dysfunction, diplopia, ischemic stroke, and hypopituitarism, with a 2-, 5- and 10-years rates of 7%, 10%, and 15%, respectively [10]. These findings stress the need for a long-term, comprehensive follow-up of post-interventional complications. Furthermore, they underline the fact that radiation therapy is not benign and should not be administered without a clear indication (progressive CN deficit or asymptomatic growing lesion). The authors also found that the larger the lesion, the higher the risk of post-SRS complications.

Correa et al. [22] showed that SRT carries similar rates of clinical and radiological improvements than SRS. In their cohort of *N* = 89 patients treated either with SRS or SRT, 7% of the patients presented transient optic neuropathy, 2% had transient trigeminal neuropathy, and one patient presented a total asymptomatic occlusion of the internal carotid artery [22].

### 5.3. Occlusion of the ICA: A Rare but Extreme Complication

**Figure 5:**
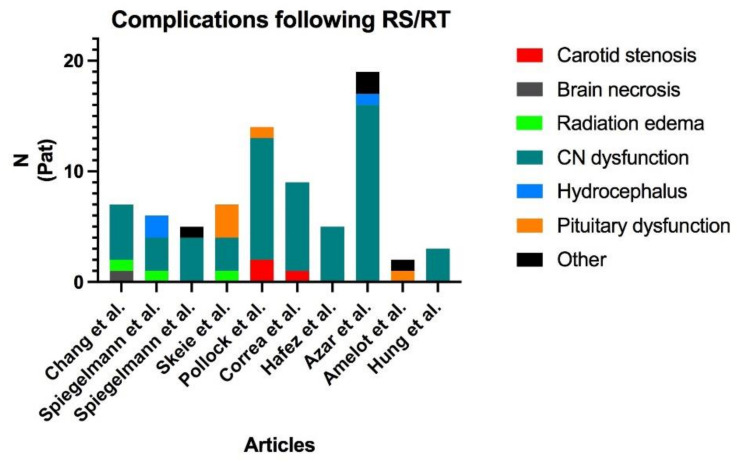
Distribution of the complications found in the review of the literature. Two publications reported no complications ([9,18]) and are, therefore, not shown in the figure. Cranial nerve dysfunction is by far the most frequent complication, and carotid stenosis is marginal. RS: Radiosurgery; RT: Radiotherapy; CN: Cranial nerves; Pat: Patients.

Despite their benign nature, their natural course tends to evolve towards CN deficits, ICA encasement, and pituitary dysfunction. SRS and SRT may relieve symptoms but carry risks in terms of local complications. As mentioned above, complications following SRS/SRT in the cavernous region are not uncommon, even though ICA occlusions and ischemic events seem to be rare. In our review, only 3 cases of ICA insufficiency were retrieved (0.03% of whole cohort, of which 2 were symptomatic), accounting for 2.5% of the overall complications (Figure 5). Recently, Graffeo et al. showed that ICA stenosis or occlusion was common after SRS for CSM, while inexistant in the case of other parasellar tumors. The authors could state that pre-interventional ICA encasement is a risk factor further post-interventional ICA stenosis or occlusion, while the risk of ischemic complications is marginal [15]. However, when these occur, the surgical strategy should be clear and elaborated on by a senior vascular neurosurgeon. This is one of the reasons why neurosurgeons must be kept aware of CSMs and should be involved in their management, regardless of the treatment planned.

Abeloos et al. reported a similar case in the late 2000. In their report, the patient presented a complete ICA occlusion contemporary to a shrinking of the CSM, after Gamma Knife treatment. In their case, the patient had no neurological deficit, and the authors recommended avoiding hot spot of dose to the intracavernous segment of the ICA [29]. Finally, Conti et al. report the occlusion of ICA following fractionated RT in a patient with a large spheno-petro-clival meningioma, raising awareness on vascular complications in other locations when it comes to large lesions encasing the ICA [30].

### 5.4. Radiation Vasculopathy in Neurosurgery

In a recent publication, Twitchell et al. present and discuss the concept of radiation vasculopathy as a complication of radiation therapy [31]. The authors present four cases similar to the case presented in this report, presenting a symptomatic vasculopathy after they underwent radiation therapy as a treatment of an astrocytoma, a spinal cordoma, an adnexal carcinoma, and a craniopharyngioma, respectively. The authors showed that vasculopathy not only results in stenosis of major cerebral vessels but may also provoke aneurysms. Other relevant reports showing that endovascular stenting and surgical clipping may sometimes be necessary, and also describe this complication, in particular in the treatment of pituitary adenomas [32,33,34].

## 6. Conclusions

Radiation therapy, be it by SRS or SRT, carry fewer complications than open surgery, with similar rates of tumor control. However, our case presented with symptomatic vascular insufficiency already two years after the initial SRS treatment, showing the importance of a follow-up of irradiated CSMs not only by a radio-oncologist but also by a neurosurgeon. This illustrates the importance of multidisciplinary management of CSMs.

## Figures and Tables

**Table 1:** Grading of the articles according to the Newcastle–Ottawa Quality Assessment Scale for quality assessment of non-randomized studies. The number of * corresponds to the number of fulfilled criteria [17].

Articles	1	2	3	4	5	6	7	8	9	10	11	12
Selection	***	***	***	***	***	***	***	***	***	***	***	***
Comparability	*	*	*	*	*	*	*	*	*	*	*	*
Outcome	**	***	***	***	***	***	***	***	***	***	***	***
Result	Fair	Fair	Fair	Fair	Fair	Fair	Fair	Fair	Fair	Fair	Fair	Fair

**Table 2:** Summary of the results of the literature search, resulting in 12 original articles reporting long-term complications after stereotactic radiosurgery or radiotherapy. GK: Gamma knife; SRS: Stereotactic radiosurgery; LINAC: Linear accelerator; Stereotactic radiotherapy; RT: Radiotherapy. Pro: Prospective; Retro: Retrospective; Pat: Patients; Complic: Complications.

*N*	Author	Year	Journal	Nature	N (Pat)	FU Duration (Months)	Technique	Carotid Stenosis	Other Complic.
1	Chang et al. [7]	1998	Stereotactic and Functional Neurosurgery	Retro.	24	45.6 (mean)	LINAC	0	Brain necrosis (1); Radiation edema (1); Trigeminal hypesthesia (4); Diplopia (1)
2	Shin et al. [18]	2001	Journal of Neurosurgery	Retro.	40	42 (median)	SRS	0	0
3	Spiegelmann et al. [19]	2002	Neurosurgery	Retro.	42	36 (median)	LINAC	0	Trigeminal neuropathy (2); Visual field defect (1); Hydrocephalus (2); Radiation edema (1)
4	Litré et al. [9]	2008	International Journal of Radiation Oncology, Biology, Physics	Pro.	100	33 (mean)	SRS	0	0
5	Spiegelmann et al. [20]	2010	Journal of Neurooncology	Pro.	102	67 (mean)	LINAC	0	Deafferentation pain (1); Facial hypesthesia (1); Visual loss (1); Neuropathy of VI (2)
6	Skeie et al. [21]	2010	Neurosurgery	Pro.	100	82 (mean)	GK	0	Optic neuropathy (2); Pituitary dysfunction (3); Worsening of diplopia (1); Radiation edema (1).
7	Pollock et al. [10]	2013	Journal of Neurosurgery	Retro.	115	89 (median)	SRS	2 ischemic strokes	Hypopituitarism (1); Diplopia (2); Trigeminal dysfunction (9)
8	Correa et al. [22]	2014	Radiation Oncology	Pro.	89	73 (median)	SRS, SRT	1 (asymptomatic)	Optic neuropath (6); Trigeminal neuroathy (2)
9	Hafez et al. [8]	2015	Acta Neurochirurgica	Retro.	62	36 (mean)	GK	0	Trigeminal dysfunction (3); Diplopia (1); Worsening of visual loss (1)
10	Azar et al. [24]	2017	Stereotactic and Functional Neurosurgery	Retro.	166	32.4 (mean)	GK	0	Worsening diplopia (3); Worsening visual impairment (2); Facial dysfunction (2); Trigeminal neuropathy (1); Unspecified (8); Adverse radiation effect (2)
11	Amelot et al. [4]	2018	Journal of Neurosurgery	Retro.	53	120 (median)	RT, GK	0	Hypopituitarism (1); Radioinduced meningioma (1)
12	Hung et al. [23]	2019	Journal of Neurooncology	Retro.	95	59 (median)	GK	0	Worsening trigeminal dysfunction (1); Worsening diplopia (1) Worsening visual loss (1)
				Total Patients	988			Total Complications	Carotid stenosis: 3 (0.3%); Brain necrosis: 1 (0.1%); Radiation edema: 3 (0.3%); CN dysfunction: 58 (5.9%); Hydrocephalus: 3 (0.3%); Pituitary dysfunction: 5 (0.5%); Others: 4 (0.4%)

## Data Availability

Not applicable.

## References

[1] Meling T.R., da Broi M., Scheie D., Helseth E. (2019). Meningiomas: Skull base versus non-skull base. Neurosurg. Rev..

[2] Graillon T., Regis J., Barlier A., Brue T., Dufour H., Buchfelder M. (2020). Parasellar Meningiomas. Neuroendocrinology.

[3] Maiuri F., Mariniello G., Guadagno E., Barbato M., Corvino S., Caro M.D.B.D. (2019). WHO grade, proliferation index, and progesterone receptor expression are different according to the location of meningioma. Acta Neurochir..

[4] Amelot A., van Effenterre R., Kalamarides M., Cornu P., Boch A.-L. (2018). Natural history of cavernous sinus meningiomas. J. Neurosurg..

[5] Sheehan J.P., Starke R.M., Kano H., Kaufmann A.M., Mathieu D., Zeiler F.A., West M., Chao S.T., Varma G., Chiang V.L.S. (2014). Gamma Knife radiosurgery for sellar and parasellar meningiomas: A multicenter study. J. Neurosurg..

[6] Milker-Zabel S., Bois A.Z.-D., Huber P., Schlegel W., Debus J. (2006). Fractionated Stereotactic Radiation Therapy in the Management of Benign Cavernous Sinus Meningiomas: Long-term experience and review of the literature. Strahlenther. Onkol..

[7] Chang S.D., Adler J.R., Martin D.P. (1998). LINAC radiosurgery for cavernous sinus meningiomas. Ster. Funct. Neurosurg..

[8] Hafez R.F.A., Morgan M.S., Fahmy O.M. (2015). Stereotactic Gamma Knife surgery safety and efficacy in the management of symptomatic benign confined cavernous sinus meningioma. Acta Neurochir..

[9] Litré C.F., Colin P., Noudel R., Peruzzi P., Bazin A., Sherpereel B., Bernard M.H., Rousseaux P. (2009). Fractionated Stereotactic Radiotherapy Treatment of Cavernous Sinus Meningiomas: A Study of 100 Cases. Int. J. Radiat. Oncol. Biol. Phys..

[10] Pollock B.E., Stafford S.L., Link M.J., Garces Y.I., Foote R.L. (2013). Single-fraction radiosurgery of benign cavernous sinus meningiomas. J. Neurosurg..

[11] Tishler R.B., Loeffler J.S., Lunsford L., Duma C., Alexander E., Kooy H.M., Flickinger J.C. (1993). Tolerance of cranial nerves of the cavernous sinus to radiosurgery. Int. J. Radiat. Oncol. Biol. Phys..

[12] Houdart E., Mounayer C., Chapot R., Saint-Maurice J.-P., Merland J.-J. (2001). Carotid Stenting for Radiation-Induced Stenoses: A report of 7 cases. Stroke.

[13] Gaudry M., David B., Omnes V., Bal L., de Masi M., Bartoli J., Piquet P.M. (2017). Radiation-induced carotid stenosis: A personnalized approach. J. Med. Vasc..

[14] Trojanowski P., Sojka M., Trojanowska A., Wolski A., Roman T., Jargiello T. (2019). Management of Radiation Induced Carotid Stenosis in Head and Neck Cancer. Transl. Oncol..

[15] Graffeo C.S., Link M.J., Stafford S.L., Parney I.F., Foote R.L., Pollock B.E. (2019). Risk of internal carotid artery stenosis or occlusion after single-fraction radiosurgery for benign parasellar tumors. J. Neurosurg..

[16] Moher D., Liberati A., Tetzlaff J., Altman D.G., Group P. (2009). Preferred reporting items for systematic reviews and meta-analyses: The PRISMA Statement. Open Med..

[17] Wells G., Shea B., O’Connell D., Peterson J., Welch V., Losos M., Tugwell P. The Newcastle-Ottawa Scale (Nos) For Assessing the Quality If Nonrandomized Studies in Meta-Analyses. http://www.ohri.ca/programs/clinical_epidemiology/oxford.htm.

[18] Shin M., Kurita H., Sasaki T., Kawamoto S., Tago M., Kawahara N., Morita A., Ueki K., Kirino T. (2001). Analysis of treatment outcome after stereotactic radiosurgery for cavernous sinus meningiomas. J. Neurosurg..

[19] Spiegelmann R., Nissim O., Menhel J., Alezra D., Pfeffer M.R. (2002). Linear accelerator radiosurgery for meningiomas in and around the cavernous sinus. Neurosurgery.

[20] Spiegelmann R., Cohen Z.R., Nissim O., Alezra D., Pfeffer R. (2010). Cavernous sinus meningiomas: A large LINAC radiosurgery series. J. Neuro-Oncol..

[21] Skeie B.S., Enger P.Ø., Skeie G.O., Thorsen F., Pedersen P.-H. (2010). Gamma Knife Surgery of Meningiomas Involving the Cavernous Sinus: Long-term follow-up of 100 patients. Neurosurgery.

[22] Correa S.F.M., Marta G.N., Teixeira M.J. (2014). Neurosymptomatic carvenous sinus meningioma: A 15-years experience with fractionated stereotactic radiotherapy and radiosurgery. Radiat. Oncol..

[23] Hung Y.-C., Lee C.-C., Guo W.-Y., Shiau C.-Y., Chang Y.-C., Pan D.H.-C., Sheehan J.P., Chung W.-Y. (2019). Gamma knife radiosurgery for the treatment of cavernous sinus meningiomas: Post-treatment long-term clinical outcomes, complications, and volume changes. J. Neuro-Oncol..

[24] Azar M., Kazemi F., Jahanbakhshi A., Chanideh I., Jalessi M., Amini E., Geraily G., Farhadi M. (2017). Gamma Knife Radiosurgery for Cavernous Sinus Meningiomas: Analysis of Outcome in 166 Patients. Ster. Funct. Neurosurg..

[25] Goldsmith B.J., Wara W.M., Wilson C.B., Larson D.A. (1994). Postoperative irradiation for subtotally resected meningiomas. A retrospective analysis of 140 patients treated from 1967–1990. J. Neurosurg..

[26] O’Sullivan M.G., van Loveren H.R., Tew J.M. (1997). The Surgical Resectability of Meningiomas of the Cavernous Sinus. Neurosurgery.

[27] Sindou M.P., Alvernia J.E. (2006). Results of attempted radical tumor removal and venous repair in 100 consecutive meningiomas involving the major dural sinuses. J. Neurosurg..

[28] Shaffrey M.E., Dolenc V.V., Lanzino G., Wolcott W.P., Shaffrey C.I. (1999). Invasion of the internal carotid artery by cavernous sinus meningiomas. Surg. Neurol..

[29] Abeloos L., Levivier M., Devriendt D., Massager N. (2007). Internal Carotid Occlusion following Gamma Knife Radiosurgery for Cavernous Sinus Meningioma. Ster. Funct. Neurosurg..

[30] Conti A., Senger C., Acker G., Kluge A., Pontoriero A., Cacciola A., Pergolizzi S., Germanò A., Badakhshi H., Kufeld M. (2020). Correction to: Normofractionated stereotactic radiotherapy versus CyberKnife-based hypofractionation in skull base meningioma: A German and Italian pooled cohort analysis. Radiat. Oncol..

[31] Twitchell S., Karsy M., Guan J., Couldwell W.T., Taussky P. (2018). Sequelae and management of radiation vasculopathy in neurosurgical patients. J. Neurosurg..

[32] Brada M., Burchell L., Ashley S., Traish D. (1999). The incidence of cerebrovascular accidents in patients with pituitary adenoma. Int. J. Radiat. Oncol. Biol. Phys..

[33] Flickinger J.C., Nelson P.B., Taylor F.H., Robinson A. (1989). Incidence of cerebral infarction after radiotherapy for pituitary adenoma. Cancer.

[34] Matsumoto H., Minami H., Yamaura I., Yoshida Y. (2014). Radiation-induced cerebral aneurysm treated with endovascular coil embolization. A case report. Interv. Neuroradiol..

